# Varying dataset resolution alters predictive accuracy of spatially explicit ensemble models for avian species distribution

**DOI:** 10.1002/ece3.4725

**Published:** 2018-12-06

**Authors:** Claire M. Curry, Jeremy D. Ross, Andrea J. Contina, Eli S. Bridge

**Affiliations:** ^1^ University Libraries University of Oklahoma Norman Oklahoma; ^2^ Oklahoma Biological Survey University of Oklahoma Norman Oklahoma; ^3^ Corix Plains Institute University of Oklahoma Norman Oklahoma

**Keywords:** data resolution, grassland birds, landscape ecology, machine learning, Oklahoma, random forest, spatiotemporal exploratory models

## Abstract

Species distribution models can be made more accurate by use of new “Spatiotemporal Exploratory Models” (STEMs), a type of spatially explicit ensemble model (SEEM) developed at the continental scale that averages regional models pixel by pixel. Although SEEMs can generate more accurate predictions of species distributions, they are computationally expensive. We compared the accuracies of each model for 11 grassland bird species and examined whether they improve accuracy at a statewide scale for fine and coarse predictor resolutions. We used a combination of survey data and citizen science data for 11 grassland bird species in Oklahoma to test a spatially explicit ensemble model at a smaller scale for its effects on accuracy of current models. We found that only four species performed best with either a statewide model or SEEM; the most accurate model for the remaining seven species varied with data resolution and performance measure.

**Policy implications:** Determination of nonheterogeneity may depend on the spatial resolution of the examined dataset. Managers should be cautious if any regional differences are expected when developing policy from range‐wide results that show a single model or timeframe. We recommend use of standard species distribution models or other types of nonspatially explicit ensemble models for local species prediction models. Further study is necessary to understand at what point SEEMs become necessary with varying dataset resolutions.

## 1 INTRODUCTION

Species distribution modeling (SDM) is a tool that uses environmental and geographic variables to predict what areas are suitable for a species and to better understand what factors constrain species’ ranges (Elith & Leathwick, 2009). SDM can also be used to predict potential impacts of climate and land use change (Beaumont, Pitman, Poulsen, & Hughes, 2007; Lipsey et al., 2015). Newer regression and machine learning techniques incorporated into SDM continue to increase prediction accuracy (Cutler et al., 2007; Elith, Leathwick, & Hastie, 2008; Elith et al., 2006; Lorena et al., 2011; Phillips, Dudík, & Schapire, 2004). One such method, Spatiotemporal Exploratory Modeling (STEM), has recently been introduced as a means of coping with variation in regional drivers. STEM uses smaller, overlapping subsets of data to generate regional predictions that are combined into an average (Fink et al., 2010). This averaging of overlapping smaller models (the model type used here is referred to as the base model) allows the local models to correctly model predictor‐response relationships that may not occur in all parts of the study area, resulting in an overall map with more accurate predictions. The ensemble technique of combining overlapping predictions can be used with almost any model type (Fink et al., 2010; Fink, Damoulas, & Dave, 2013), and can cover continent‐ to hemisphere‐wide scales (Fink et al., 2018, 2013). Unfortunately, these spatially explicit ensemble models (SEEMs) are computationally expensive, because instead of predicting just one map they must predict numerous supporting maps followed by averaging them to create the final model. Additionally, the relative increase in accuracy has not been compared to the relative expense of computational time nor have SEEMs been tested at scales at which much species management occurs, such as state or regional initiatives (Brennan, Kuvlesky, & Morrison, 2005).

Spatiotemporal Exploratory Models have been developed for continental‐scale analyses because such a broad scale provides enough habitat and climate variation to require such a model. However, there are cases in which even a regional scale dataset can provide a wide range of bioclimatic heterogeneity relative to the study area, with variation in spatial and temporal processes at scales intermediate to the study area and predictor resolution, and therefore, can be suitable for this application (Johnston et al., 2015; Zuckerberg, Fink, La Sorte, Hochachka, & Kelling, 2016). The state of Oklahoma in the United States (US) provides such case because of its high biodiversity, ranking 9th for bird species richness, 15th for total species richness, and above the median in species richness for reptiles, amphibians, freshwater fish, vascular plants, and mammals in the United States. (Stein, 2002). In particular, the grassland birds of Oklahoma inhabit diverse grassland types and climatic extremes. The open habitats of Oklahoma, which contains over a third of its land area as grasslands and an additional 15% as croplands (Diamond & Elliott, 2015), contain grassland birds characteristic of habitats ranging from southeastern pine savannahs to tallgrass, mixed‐grass, and shortgrass prairies (Askins et al., 2007; Diamond & Elliott, 2015). Grassland species in areas half the size of Oklahoma in a single ecoregion have shown spatial and temporal differences in variable importance (Ethier, Koper, & Nudds, 2017). Forest species, which likewise occupy a single habitat type, also show spatial and temporal variation in predictor importance (Zuckerberg et al., 2016). Similarly, such a technique has been used on shorebirds in habitats with structural similarity to grasslands at a statewide scale (Johnston et al., 2015). Finally, Oklahoma occurs on a strong east–west climatic gradient (Oklahoma Climatological Survey, 2017) that has had profound impacts on the ecosystems of the region (Kukal & Irmak, 2016; Seager et al., 2018). Physiological balances in animals can change in importance with other environmental variables (Kearney, Simpson, Raubenheimer, & Kooijman, 2013); therefore, variable importance may be expected to change for at least some species across climatic gradients. Oklahoma's grassland habitats, agricultural importance, and susceptibility to climate change (Loarie et al., 2009; National Assessment Synthesis Team (U.S.), 2001) make it an ideal and important region to test relative efficacy of different methods for modeling species distributions.

Grasslands are one of the world's most endangered ecosystems, with declines of 82.6%–99.9% of tallgrass prairie, 30%–99.9% of mixed‐grass prairie, and 20%–85.8% of short‐grass prairie in the plains states and provinces of North America (Samson & Knopf, 1994), and as such could benefit from increased knowledge of distributional drivers. Drivers of decline include land use conversion via agriculture and changes in fire and grazing regimes (Samson, Knopf, & Ostlie, 2004), although specifics vary by region (Askins et al., 2007). The already tenuous status of grassland birds is further threatened by conversion to new crops resulting in permanent land use changes (Wright & Wimberly, 2013), generational changes in land use (Higgins, Naugle, & Forman, 2002), changes in conservation programs for grassland habitats (Klute, Robel, & Kemp, 1997), alterations to vegetation (Alward, 1999) and ecosystem structure (Brown, Valone, & Curtin, 1997; Hamer, Flather, & Noon, 2006), and climate change (McCarty, 2001). Grassland bird species are declining faster than other groups of birds (Askins et al., 2007; Hill, Egan, Stauffer, & Diefenbach, 2014; Knopf, 1994) and continue to be imperiled by ongoing and expanding threats to their habitat. Range‐wide species distribution predictions have been made for grassland birds but some species with smaller ranges are not accurately modeled (O'Connor, Jones, Boone, & Lauber, 1999), perhaps because some drivers of distribution vary regionally (Askins et al., 2007; Bakker, Naugle, & Higgins, 2002; Ethier et al., 2017), at a scale smaller than the study region. Additionally, spatial and temporal variation in habitat needs and selection pressures (Davis, 2005; Winter, Johnson, & Shaffer, 2005) or interactions with weather events (Pipher, Curry, & Koper, 2016) are known to be important in grassland birds; therefore, they are particularly suitable as a testing ground for a spatially explicit approach to modeling.

The objectives of our study were threefold. First, we estimated the distribution of Oklahoma grassland birds to understand current distribution statewide with standard species distribution modeling methods. Next, these statewide current distribution predictions allowed us to compare the statewide species distribution model for each species with SEEMs to evaluate whether this approach is suitable at the scale of our region. Finally, we compared each approach's accuracy when using fine‐ or coarse‐resolution predictor sets. Although our approach is at a smaller scale than originally envisioned for SEEMs, it is important to test their potential applicability at the smaller scales at which most management decisions are made. Our results will allow others to make decisions on whether increased accuracy in modeling is worth the additional computational effort required by newer modeling techniques and provide guidance for future work into where given modeling applications are useful.

## 2 METHODS

### 2.1 Study area

Oklahoma contains diverse vegetation and climate, making it a suitable region to examine effects of spatially explicit models. There are ca. 165 vegetation types (based on soil and vegetation composition) in 15 land cover types (Diamond & Elliott, 2015), with over a third of the vegetation in grasslands. Rainfall and temperature vary across the state (Oklahoma Climatological Survey, 2017), with annual precipitation ranging from ~43 cm of rain in the northwest to 142 cm in the southeast and mean annual temperature ranging from ~13°C in the northwest to ~17°C in the southeast. Summer temperatures over 32°C can occur from 60–115 days out of the year varying statewide. Agriculture in Oklahoma is dominated by livestock ranching and row crops (USDA/NASS, 2016) and accounted for over $2.8 billion of the state's gross domestic product in the study years (US Bureau of Economic Analysis,^2016^); Oklahoma ranks in the top 5 of US acreage for grain wheat and forage land for hay (USDA/NASS, 2016).

### 2.2 Bird surveys

We collected data 1–4 times each at 339 8‐min roadside point counts (0.13 hr) and at 87 nonroadside transects 0.3–3.1 hr and 0.3–4.3 km long (mean±SD: 1.2 ± 0.6 hr and 1.8 ± 0.8 km). Each survey was conducted stationary (point counts) or walking at an even pace (transects). We had 14 observers total (6 in 2013 and 8 in 2014). We only used sightings within 500 m of the observer to preserve identification accuracy and recognize that detection is imperfect; however, all models compared use similar data and as such it should not impact our comparison of models. A zero (absence) or 1 (presence) was assigned for each combination of date and time and species. We focused on 10 species of grassland birds found during our general surveys [Northern Bobwhite (*Colinus virginianus*); Upland Sandpiper (*Bartramia longicauda*); Horned Lark (*Eremophila alpestris*); Cassin's Sparrow (*Peucaea cassinii*); Field Sparrow (*Spizella pusilla*); Lark Sparrow (*Chondestes grammacus*); Grasshopper Sparrow (*Ammodramus savannarum*); Dickcissel (*Spiza americana*); Eastern Meadowlark (*Sturnella magna*); and Western Meadowlark (*Sturnella neglecta*)], plus the obligate brood parasite Brown‐headed Cowbirds (*Molothrus ater*) for which presence often depends on land use factors (Benson, Chiavacci, & Ward, 2013), for a total of 11 species. Many of these species are declining at the state or North American level; none are increasing in population (Sauer et al., 2017).

**Figure 1:**
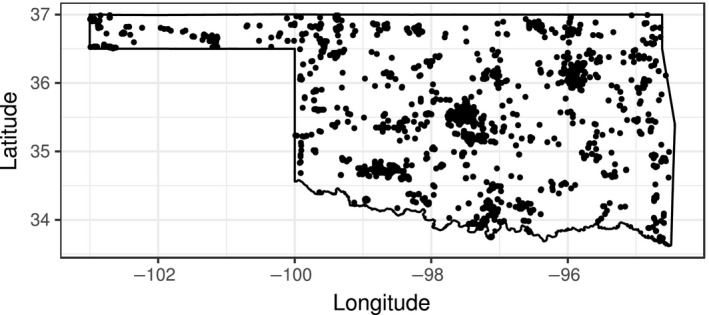
The complete dataset used in this study from eBird and surveys by the authors in 2013 and 2014 in the central U.S. state of Oklahoma in the Great Plains. The dataset was sampled such that half each was used for model training and model evaluation

We supplemented our survey data for the 11 focal species with citizen science data from the eBird Reference Dataset (Munson et al., 2014) during the months of April, May, June, and July, to match the surveys we conducted. We used complete primary checklist data from 2013–2014 and excluded casual counts. Complete checklists contain all birds sighted by the observer; primary checklists are the main checklist submitted when more than one observer submitted checklists for the same observations. We restricted use of eBird samples to ≤4.3 km and ≤3.1 hr to be comparable to our surveys. We used the point count center or the transect midpoint as the count location for our surveys to have comparable precision to eBird coordinates (Fink et al., 2010). Likewise, because some eBird sightings will be from similar locations, we used all replicates of our point counts and transects. Because some of our observers entered sightings from before and during our surveys into eBird, we eliminated 14 counts from 2013 and 2014 that were within two hours of the actual survey start time and within 15 km of the survey start location. The combined dataset contained 5,422 complete checklists (158 transect sampling events, 613 point count sampling events, and 4,651 eBird sampling events). Data points are shown in Figure 1.

To partition training and evaluation datasets, the combined dataset was split randomly for each species using the createDataPartition function in the CARET package (Kuhn, 2017), which samples such that both training and evaluation splits have similar distributions of presence and absences.

### 2.3 Predictors

We used bioclimatic variables from WorldClim at 30‐s resolution (Hijmans, Cameron, Parra, Jones, & Jarvis, 2005), conservation easement status (O'Connor et al., 1999), and land cover variables (USDA/NRCS ‐ National Geospatial Center of Excellence, 2011) to predict bird distribution (Table S1). We also included effort (length of observation in distance and time) and time of day in the analysis to control for differences in bird activity and observer effort that may influence species checklists. Neighborhood predictors were calculated by the values in rectangular areas around each point, at the scale of 5 × 5 pixels (150 × 150 m) and 15 × 15 pixels (450 × 450 m) (Fink et al., 2010). Although the 15 × 15 pixel unit is smaller than our 500 m cutoff, most sightings are from even larger areas with the maximum length being under 4.3 km, an area comparable to Fink et al., 2010. Additionally, using a neighborhood value centered at the location point still provides information about the neighborhood, whether or not it overlaps or surrounds the sighting. We looked at proportion of each land cover class and proportion of summed open space land covers (grasslands, hay/pasture, cropland, herbaceous wetlands, and barren land) since grassland bird occupancy can be influenced by the total nonstructural cover (McDonald, 2017). Neighborhoods were created in QGIS 2.16 with the GRASS r.neighbors processing tool (Quantum GIS Development Team, 2016).

We tested for the effects of using coarser (lower resolution) rasters to see if matching predictor and response variable scale affected accuracy. This is applicable as lowering raster resolution could be a route to making potentially more accurate models available to more researchers and managers. We scaled our previously created predictor rasters from their native or previously resampled 30 m resolution to the approximate scale of our largest response data resolution, by decreasing cell size 144‐fold to 4.32 km using means in the “aggregate” function in the R package Raster (Hijmans, 2016). Using these coarser predictor sets trimmed, the 2013–2014 dataset slightly down to 5,327 checklists (2,664 for training and 2,663 for evaluation).

### 2.4 Species distribution models

We ran models on Amazon Web Services (AWS) Elastic Cloud Computing (EC2) m4.4xlarge instances (16 vCPU and 64 GiB memory).

#### 2.4.1 Base model

To create all species distribution models, we used random forest regression trees (Breiman, 2001) in the R package randomForest (Liaw & Wiener, 2002). Random forest gives results competitive to those from other machine learning techniques such as boosted regression trees and bagged decision trees (used in Fink et al., 2010 for the nonspatially‐explicit comparison model). Minimal tuning parameters are required (Caruana & Niculescu‐Mizil, 2006; Cutler et al., 2007; Guo, Graber, McBurney, & Balasubramanian, 2010). Random Forests are suitable for species distribution models (Lorena et al., 2011; Prasad, Iverson, & Liaw, 2006) even with few presence records (Mi, Huettmann, Guo, Han, & Wen, 2017). The random forest algorithm bootstraps a subset of the data using only a set proportion of the predictor variables. It then calculates the error rate on training data using the “out of bag” sample (the portion of data not used in the bootstrap for each tree) (Hastie, Tibshirani, & Friedman, 2001). The trees are then averaged for a final model (Prasad et al., 2006). All random forests (both support set and statewide models) were generated with 500 trees which are generally suitable to achieve stability and accuracy (Cutler et al., 2007). We used the default number of variables per bootstrap tree (default “mtry” = the square root of the number of predictor variables) for all trees because this is known to result in accurate predictions (Cutler et al., 2007).

Maps were created using the predict function in RASTER at the resolution of the original predictor datasets (30 m and 4.32 km). For the maps, we assumed a uniform effort and time of day by creating constants for prediction: mean effort (distance and time) and time of day rasters. Thus, all predicted distribution models are generated assuming survey effort does not vary geographically and survey effort is typical for both surveys and citizen science efforts in 2013 and 2014 (mean time: 0.73 hr; mean distance: 0.75 km). The time of day raster for prediction was given a value of 7:00 a.m. (Fink et al., 2010). Prediction values for evaluation did not use these constants.

#### 2.4.2 Statewide and SEE models

We created four models for each species at varying spatial scales: a statewide model and three SEEMs. The statewide model allowed us to compare the performance to SEEMs. A random forest model was created for the statewide scale for each species using all training data. The three remaining models are at varying support set scales, with some modifications from Fink et al. (2010). First, the scale of our support sets reflects the state extent (i.e., our small, medium, and large scales are relatively smaller than those needed for a continent‐wide scale). As our survey goals are to determine breeding distribution only, we used a broader temporal window (April‐July in all years) for our model. Secondly, for all base models, we used random forest classification trees (Breiman, 2001) as described above. Finally, our geographic sampling of the training and evaluation datasets, described in more detail in the next paragraph, reflects the differing nature of our base models. Fink et al. (2010) sampled 63% of each support set to imitate bootstrapping sampling, but we used the full data set for each support set region.

**Figure 2:**
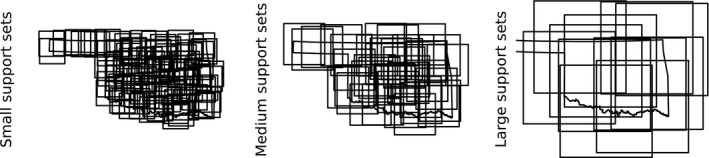
Support sets of small (left), medium (middle), and large (right) scale overlaid over the study area of Oklahoma, USA

Building a SEEM consists of creating random support sets, generating trees and predictions for each support set, and then, combining each support set model predictions into the final overall prediction. We created stratified random points in the study area to create support sets (Figure 2). The randomization of the support set center is important to fit ensemble models with low bias and high variance (Kuncheva & Whitaker, 2003). We used the “spsample” function from the R package SP (Bivand, Pebesma, & Gomez‐Rubio, 2013; Pebesma & Bivand, 2005) and created squares of size small (100 boxes of 120 × 120 km), medium (37 boxes of 200 × 200 km), or large (12 boxes of 450 × 450 km) around these points, which resulted in no significant difference in pixel coverage (*F*_2,147_ = 0.63, *p* = 0.53; small mean: 6.9, median 7, range 2–10; medium mean: 6.3, median 7, range 2–11; large mean: 6.6, median 7, range 2–10) before removing support sets with too few (<25) or uniform (all presence or all absence) checklists (models cannot run with uniform values). Using a larger number of base model pixel coverage is ideal to reduce “blockiness” in final ensemble maps and prediction coverage, but we were limited by computational costs. Each support set included all checklists from the training dataset located within its boundaries. All remaining support set rasters for a given scale (small, medium, or large) were combined into one larger raster using the RASTER mosaic() function to get the mean value of each pixel (ranging from 0 to 1), creating the spatially explicit ensemble (Fink et al., 2010; Hastie et al., 2001; Oppel et al., 2012) made of regional random forest predictions. This process was repeated at the three spatial scales, resulting in three SEEMs per species.

#### 2.4.3 Model evaluation and error

To evaluate model performance, we created a statewide grid of 10 × 10 km cells and randomly sampled no more than 10 observations from each grid cell for spatial uniformity (Fink et al., 2010) using the held back data. The actual presence or absence from each checklist is compared to predicted values at each cell with the date and time of the sighting (instead of the uniform date and time used to create the maps). These sampling grid cells are larger than either predictor size and are used to ensure that we do not weight the accuracy of the models toward regions with more reports or surveys. We repeated the evaluation sampling 50 times to create a performance distribution for each model and error type (Fink et al., 2010). We noted the scale (small, medium, large, statewide) with best performance measures for each species and compared performance with notched box plots (Chambers, Cleveland, Kleiner, & Tukey, 1983).

Performance measures were root mean square error (RMSE) and area under the curve (AUC). RMSE is calculated from the model residuals, taking the squared value of observed minus expected values, then taking the square root to return to original units; a larger value indicates the model deviates further from expected (Kuhn & Johnson, 2013). AUC is a summary of model performance measuring how often the model misclassifies individual test observations; AUC ranges from 0 to 1, with 1 being perfect and 0.5 being a model that performs no better than random chance (Hanley & McNeil, 1982; James, Witten, Hastie, & Tibshirani, 2013).

To compare computing efficiency, we used the R package MICROBENCHMARK to measure runtimes. All runtimes included randomForest trees and RASTER prediction; ensembles also included mosaic creation time. We compared runtimes with a ratio of scaled model runtime to statewide model runtime as computational times will differ by the user's available machines.

## 3 RESULTS

Current statewide distributions are shown in panel (a) of Figures 3, 4, 5, 6 and Supporting information Figures S1–S7. SEEMs took 2.7–12.7 times longer (with fine resolution predictors) or 2.6–22.7 times longer (with coarse resolution predictors) to run than a statewide model, depending on species.

**Figure 3:**
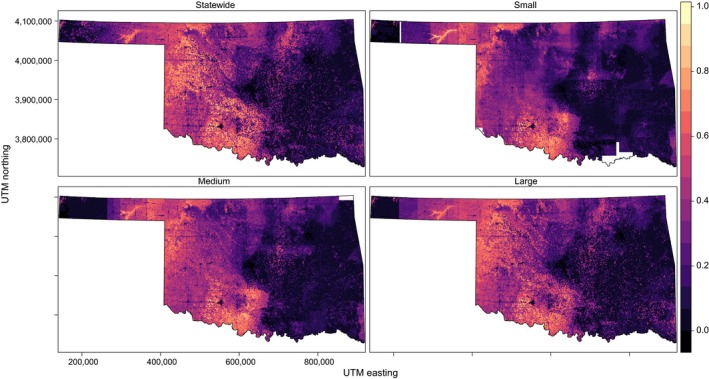
Species distribution model for Northern Bobwhite generated at four scales (statewide and three spatially explicit ensemble models at large, medium, and small support set sizes) with 30 m resolution in Oklahoma. Color scale indicates probability of occurrence from 0 to 1. Blank areas (in white within the state boundaries) were not able to calculate a model

**Figure 4:**
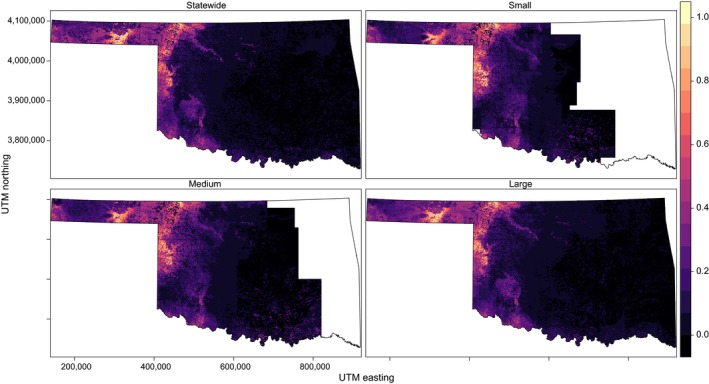
Species distribution model for Cassin's Sparrow generated at four scales (statewide and three spatially explicit ensemble models at large, medium, and small support set sizes) with 30 m resolution in Oklahoma. Color scale indicates probability of occurrence from 0 to 1. Blank areas (in white within the state boundaries) were not able to calculate a model

**Figure 5:**
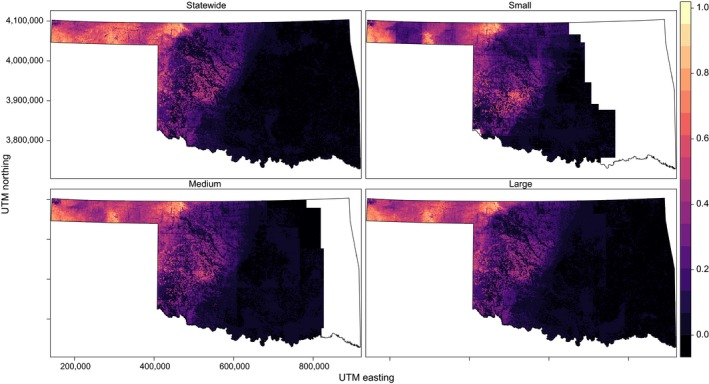
Species distribution model for Western Meadowlark generated at four scales (statewide and three spatially explicit ensemble models at large, medium, and small support set sizes) with 30 m resolution in Oklahoma. Color scale indicates probability of occurrence from 0 to1. Blank areas (in white within the state boundaries) were not able to calculate a model

**Figure 6:**
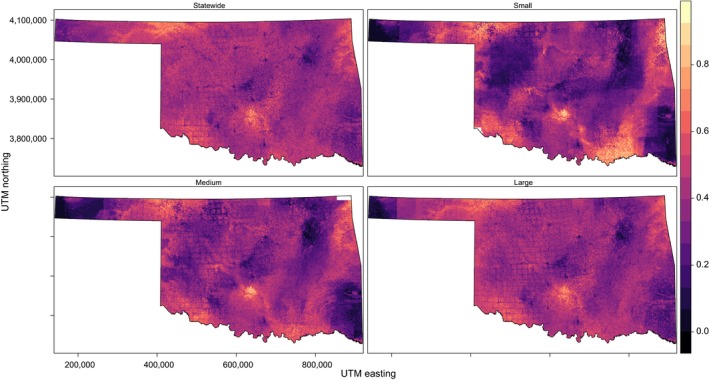
Species distribution model for Brown‐headed Cowbird generated at four scales (statewide and three spatially explicit ensemble models at large, medium, and small support set sizes) with 30 m resolution in Oklahoma. Color scale indicates probability of occurrence from 0 to 1. Blank areas (in white within the state boundaries) were not able to calculate a model.

**Figure 7:**
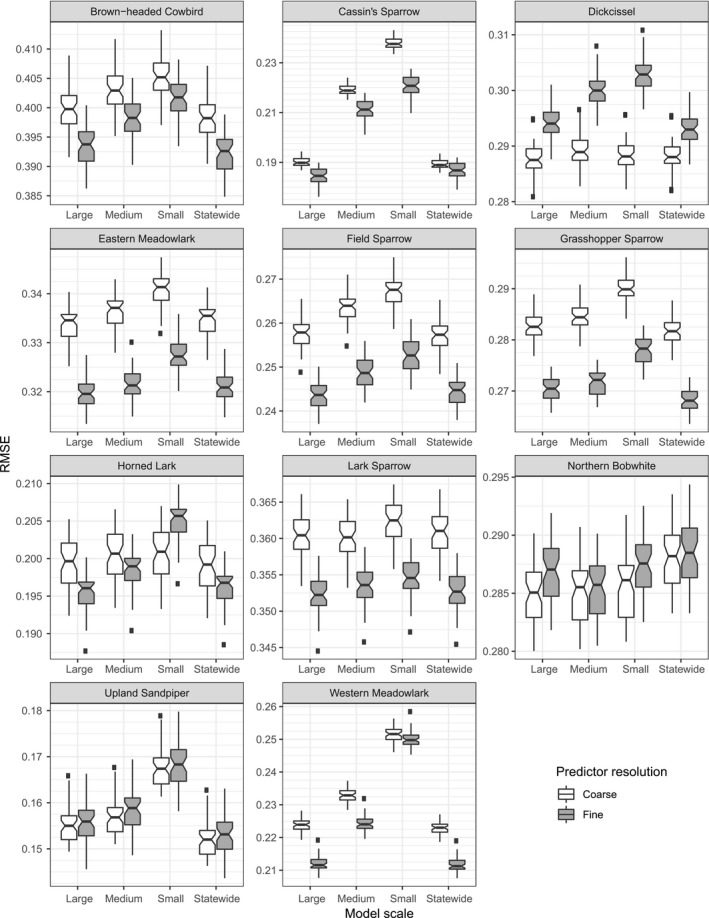
RMSE evaluations for all 44 models compared by predictor resolution. Each panel shows one species. Overlapping notches on boxplots show no difference; nonoverlapping notches show a significant difference in medians. Centerline represents median. Fine grid lines are shown to facilitate notch comparison

**Figure 8:**
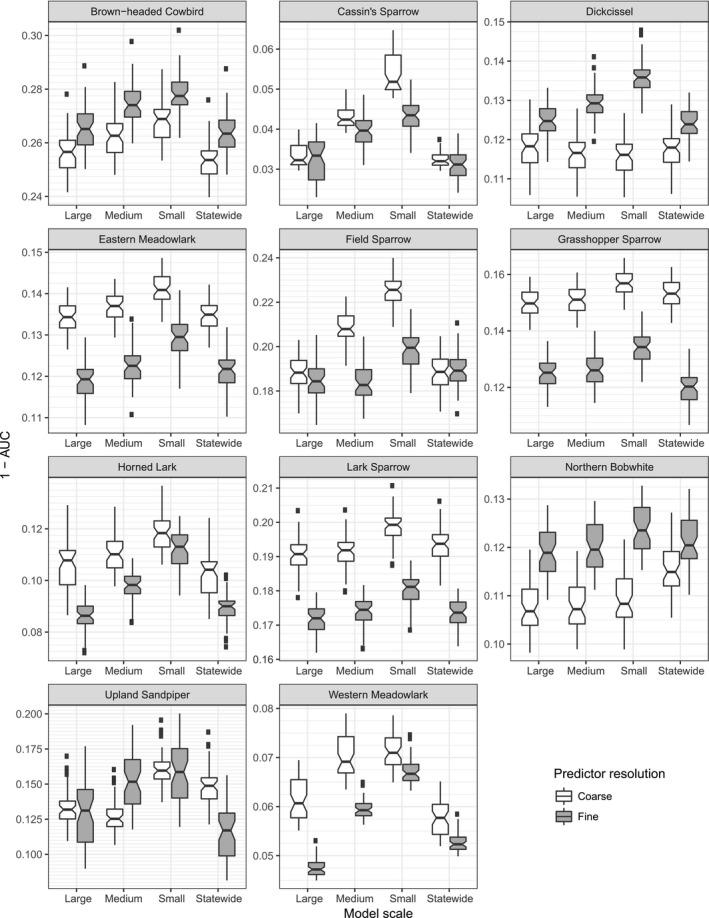
AUC evaluations for all 44 models compared by predictor resolution. Each panel shows one species. AUC = 0.5, where prediction is random, and above which prediction is better than random. We show the y‐axis as 1—AUC so that a lower value is better prediction to facilitate comparison with RMSE in Figure 7 . Overlapping notches on boxplots show no difference; nonoverlapping notches show a significant difference in medians. Centerline represents median. Fine grid lines are shown to facilitate notch comparison

Spatially explicit ensemble models outperformed statewide models for only Northern Bobwhite and Western Meadowlark within each data resolution for both AUC (Figure 7) and RMSE (Figure 8). Statewide models outperformed or equaled SEEMs within each data resolution for Brown‐headed Cowbird and Dickcissel for both AUC and RMSE.

Coarse resolution models consistently outperformed fine resolution models in both AUC and RMSE for Dickcissel. Fine resolution models consistently outperformed coarse resolution models in both AUC and RMSE for Lark Sparrow, Grasshopper Sparrow, and Eastern Meadowlark.

The remaining species’ best model (statewide or a SEEM) differed between resolutions or with choice of error evaluation.

## 4 DISCUSSION

Although SEEMs increase model accuracy over continental scales (Fink et al., 2013, 2010), our study found their performance differed by species and predictor resolution even in a state with variable climate and diverse ecoregions. Two species were often better represented by SEEMs, suggesting their distributional processes may vary regionally. There were few obvious commonalities among these species that would lead to SEEMs being more accurate for them. One species is nonpasserine (Northern Bobwhite), and the other is a common grassland passerine (Western Meadowlark). Two species were always better with statewide models (Brown‐headed Cowbird and Dickcissel). The cowbird is strongly dependent on habitat structure (Benson et al., 2013; Bernath‐Plaisted, Nenninger, & Koper, 2017), but these variables are not what is measured by the predictor layers that we used. Dickcissel is known for its semi‐nomadic movement patterns (Temple, 2017); as such, neither species may be as dependent on local climatic variation mapped by the BioClim predictor inputs. The inconsistencies in the remainder of the species suggest that a larger sample of species and predictor resolutions is needed to compare why models are appropriate for given situations. On our original models, the predictors are consistently finer‐scaled (30 m) than some, but not all, response location data (ranging from exact point count locations to aggregate sightings along a 4.3 km transect). However, Fink et al. (2010) used transects almost twice as long as ours (up to 8.1 vs. 4.3 km) with 30 m resolution predictor data, so that should not account for differences between our results.

A potential mechanism for variation between species includes whether species’ distributions depend more upon bioclimatic versus ecological variables, as bioclimatic variables should change more smoothly over a larger area (potentially reducing the need for adaptive local models). It could also be that species‐specific processes determine whether SEEMs are required. However, one benefit of random forest models and other machine learning methods is minimal tuning and expert opinion required to generate an accurate map (Fink et al., 2010). Requiring researchers to choose spatial scale based on expert opinion of variable importance negates this benefit. However, the fact that most species showed different model performance based on whether we used fine or coarse predictor resolution suggests that model performance depends at least partially on dataset resolutions. Researchers who suspect that a SEE model is appropriate for their dataset and system can compare a small number of base models for different regions or times and see if relationships vary among the test models.

An alternative approach for modelers seeking increased accuracy is the use of nonspatially explicit ensemble models, where different base models (predicting for the whole study area) are combined to produce a single prediction map (Araújo & New, 2007; Oppel et al., 2012). We recommend this approach as more efficient for regional managers. Multiple maps will still be generated for the whole study area (*n* = number of base models used), but typically fewer than the number of support sets created in a SEEM or STEM. These types of ensembles are known to increase accuracy relative to a single base model (Araujo & New 2007; Oppel et al., 2012). Although large‐scale solutions to conserve grasslands are needed (Samson et al., 2004), local and regional conservation and management efforts also have critical impacts (Brennan et al., 2005). We expected that SEEMs would be most accurate and therefore relevant to wildlife management in this state with diverse ecotypes that occur at scales larger than predictors but smaller than our study region. However, based on our study, we recommend that when using a single base model type, all distribution model types should be run (statewide and at least one or more scales of SEEM) if computing capacity is available.

Accurate species distribution models can help us understand what factors, both environmental and land use, drive species declines (Elith & Leathwick, 2009), but we need to conduct modeling with predictors and responses at the appropriate spatial scale. Further research is needed to elucidate at what study scale and data resolution SEEMs become appropriate. In fact, we found a modern laptop or desktop unable to handle fine resolution SEEMs and turned to cloud computing to complete them, so the length of time and computing expense involved can be substantial. Coarser predictor models were much quicker to run (less than an hour of increase relative to statewide models on the high‐speed cloud computing), but they were still many times longer in runtime than the comparable statewide model. At the continental and temporally fine‐grained scales, Fink et al. (2010)’s result still stands; it is at intermediate scales where more research is needed.

## AUTHORS' CONTRIBUTIONS

ESB, JDR, AJC, and CMC conceived the ideas and designed methodology. ESB and JDR collected data. CMC analyzed the data. All authors contributed critically to the drafts and gave final approval for publication.

## DATA ACCESSIBILITY

Model code and survey data are available at https://doi.org/10.5061/dryad.7m13q9b. eBird data are available from eBird.org (Munson et al., 2014).
