# Supplementary figures and images for: Varying dataset resolution alters predictive accuracy of spatially explicit ensemble models for avian species distribution
Source: Ecol Evol. 2018 Dec 6;8(24):12867–78. doi: 10.1002/ece3.4725 (PMC6308883; doi:10.1002/ece3.4725)

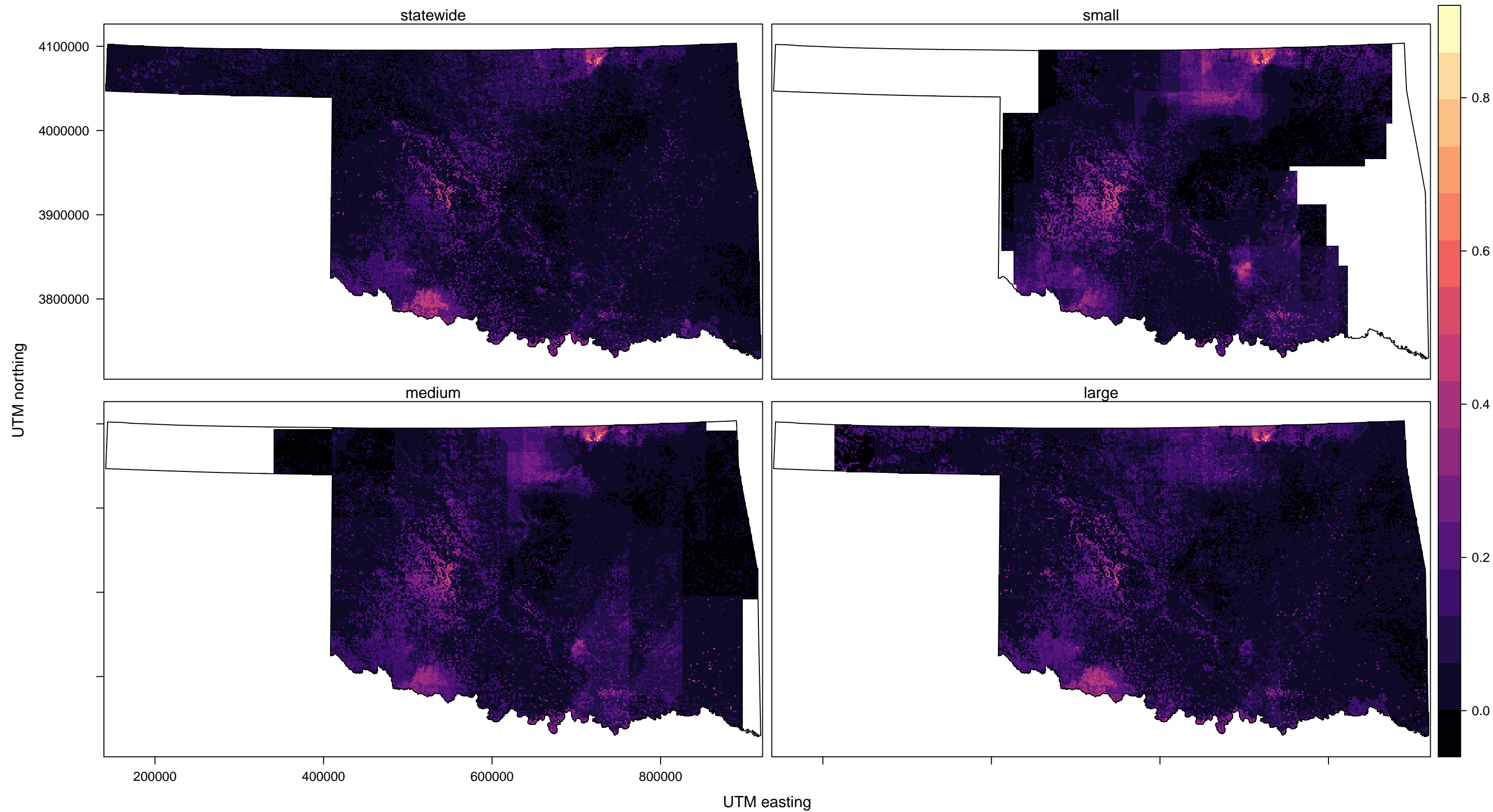

Supplement: Supplementary file 1 [file ECE3-8-12867-s001.pdf]

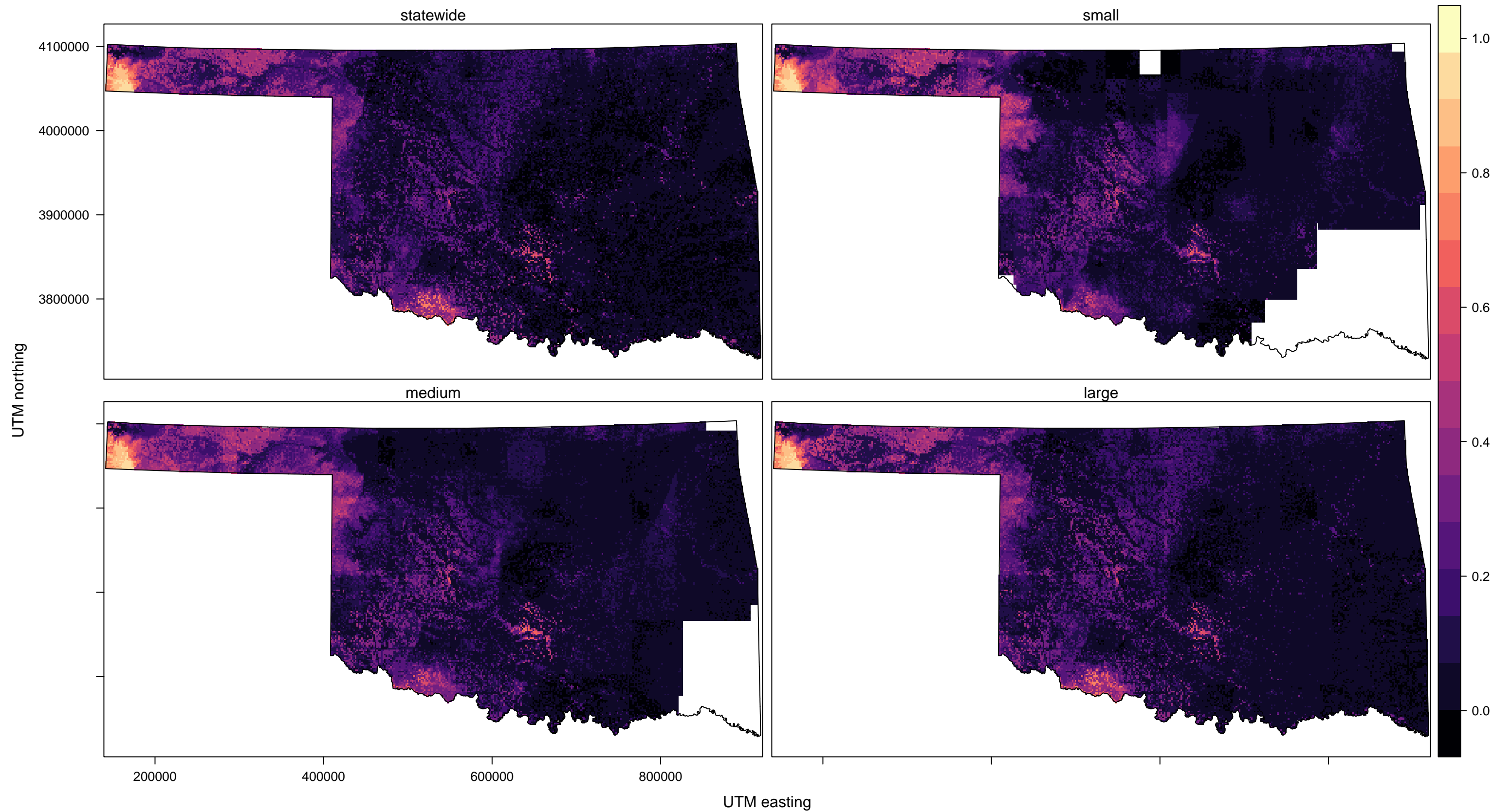

Supplement: Supplementary file 2 [file ECE3-8-12867-s002.pdf]

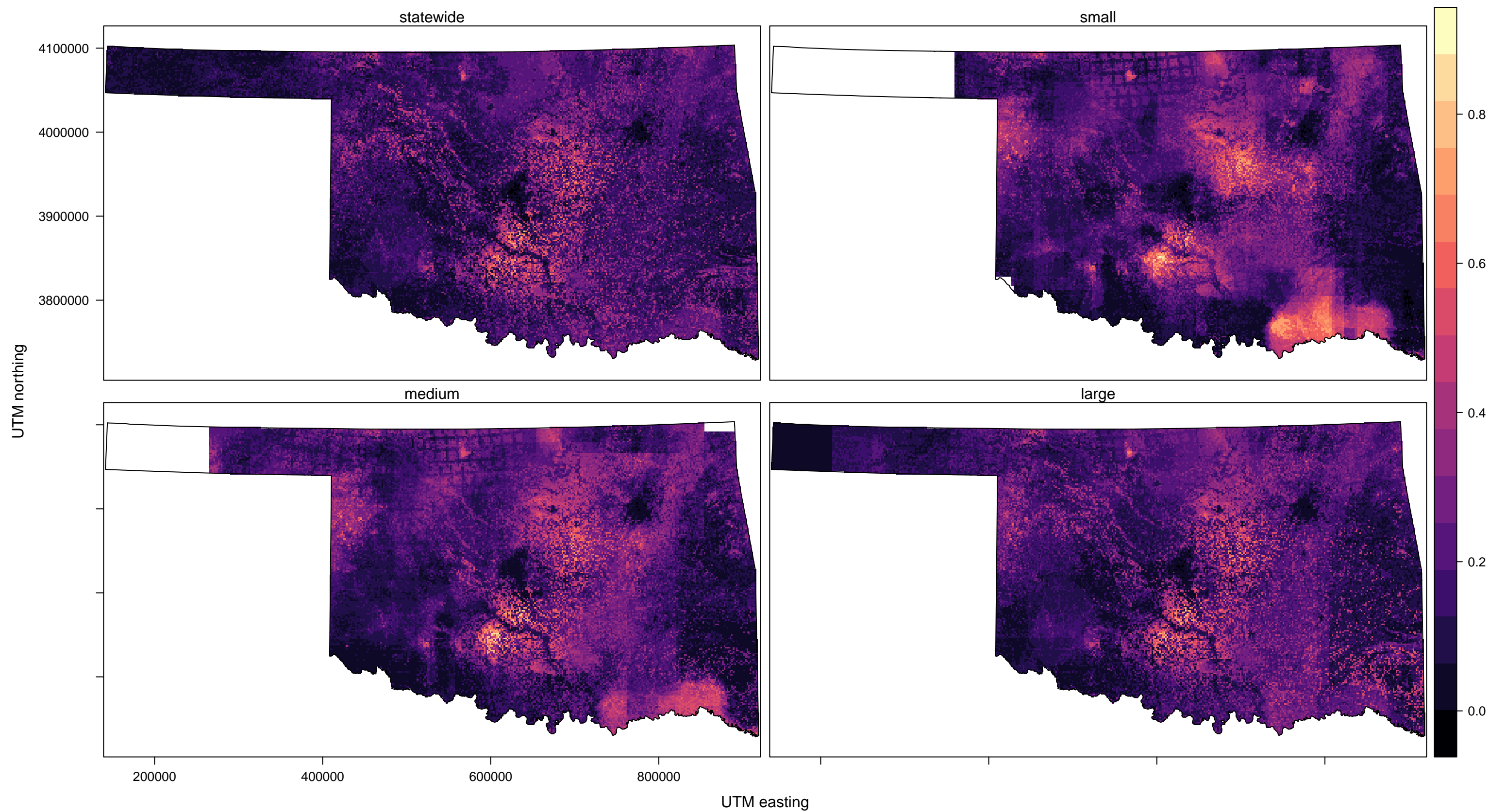

Supplement: Supplementary file 3 [file ECE3-8-12867-s003.pdf]

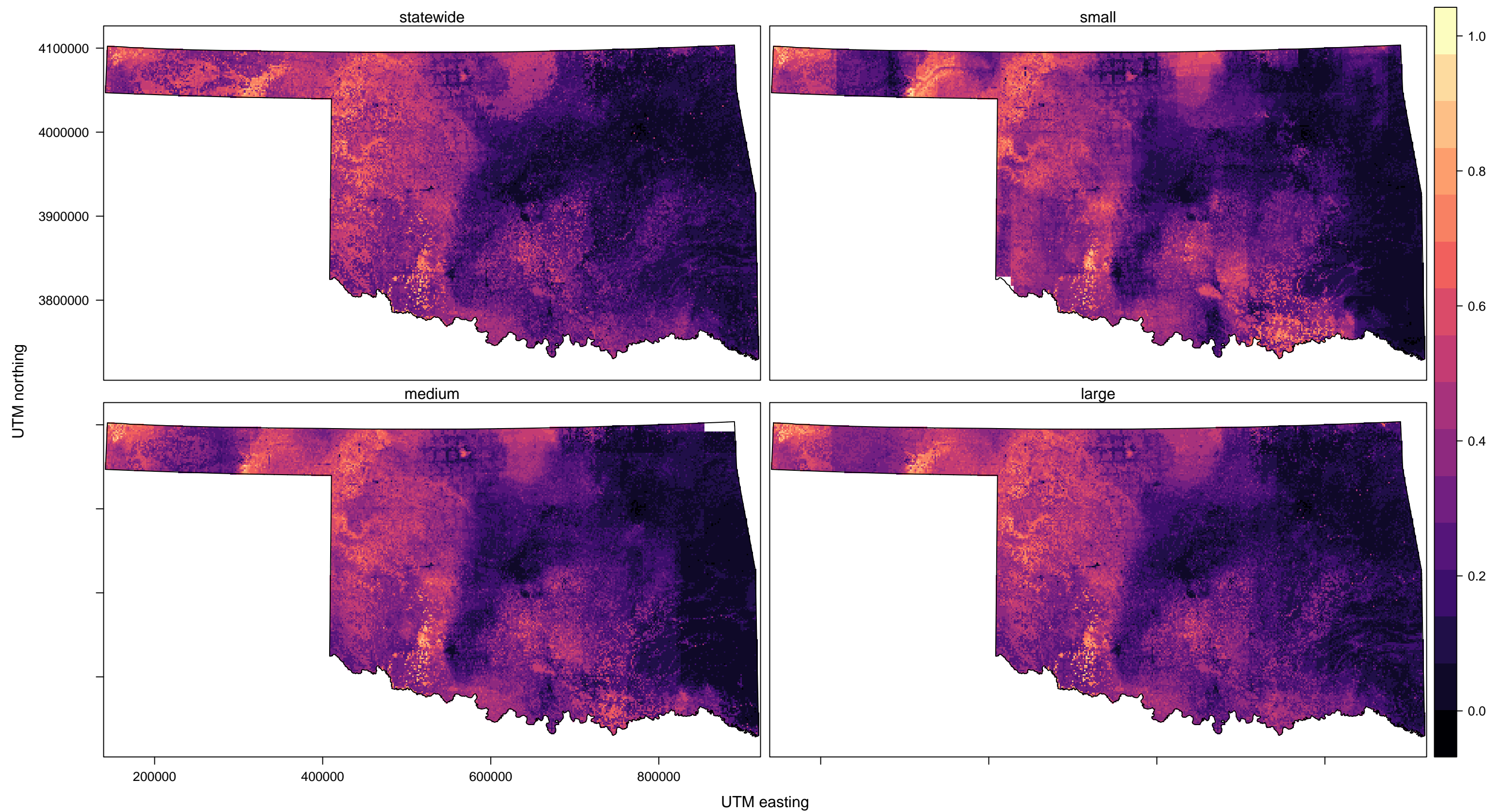

Supplement: Supplementary file 4 [file ECE3-8-12867-s004.pdf]

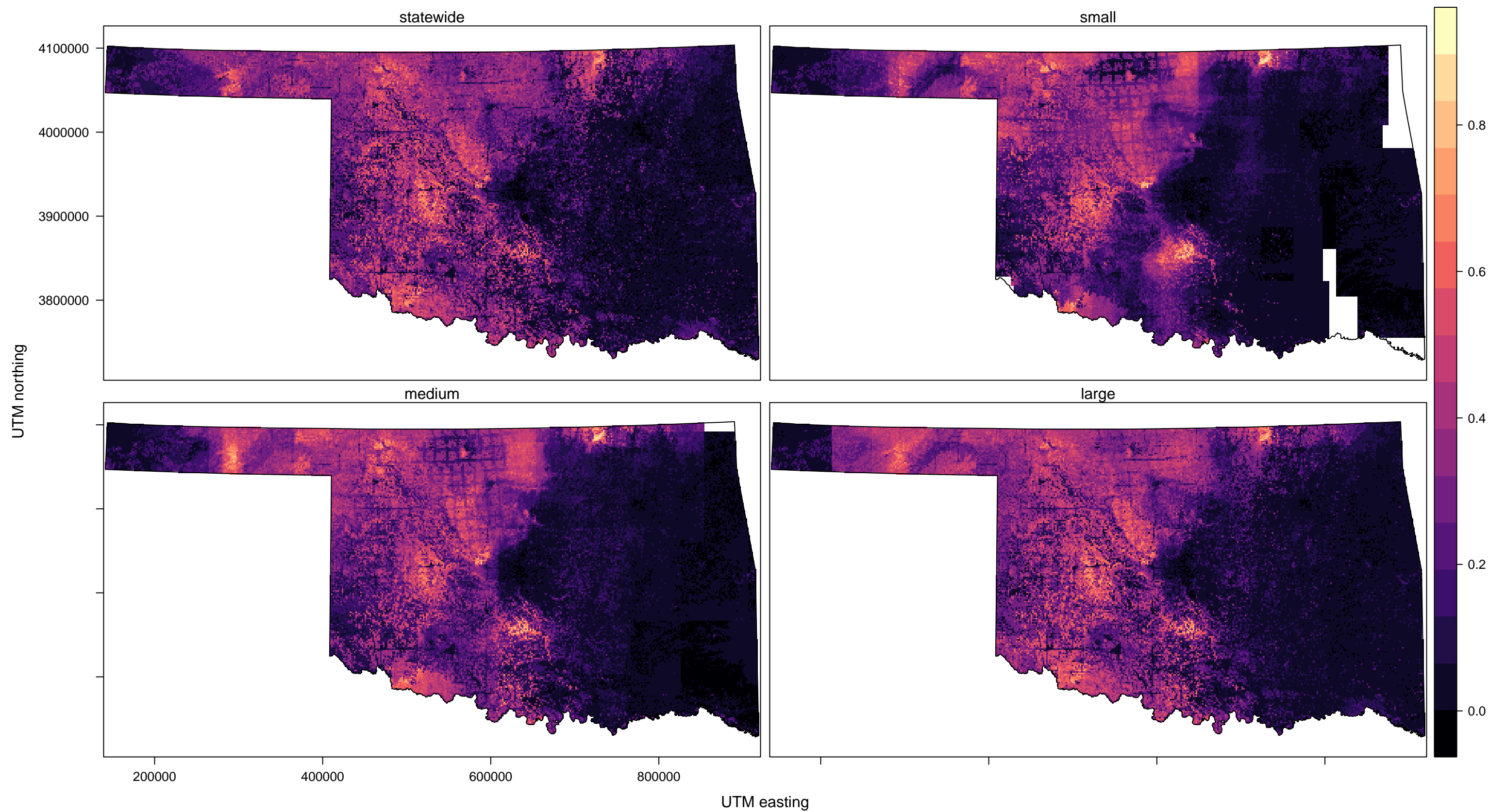

Supplement: Supplementary file 5 [file ECE3-8-12867-s005.pdf]

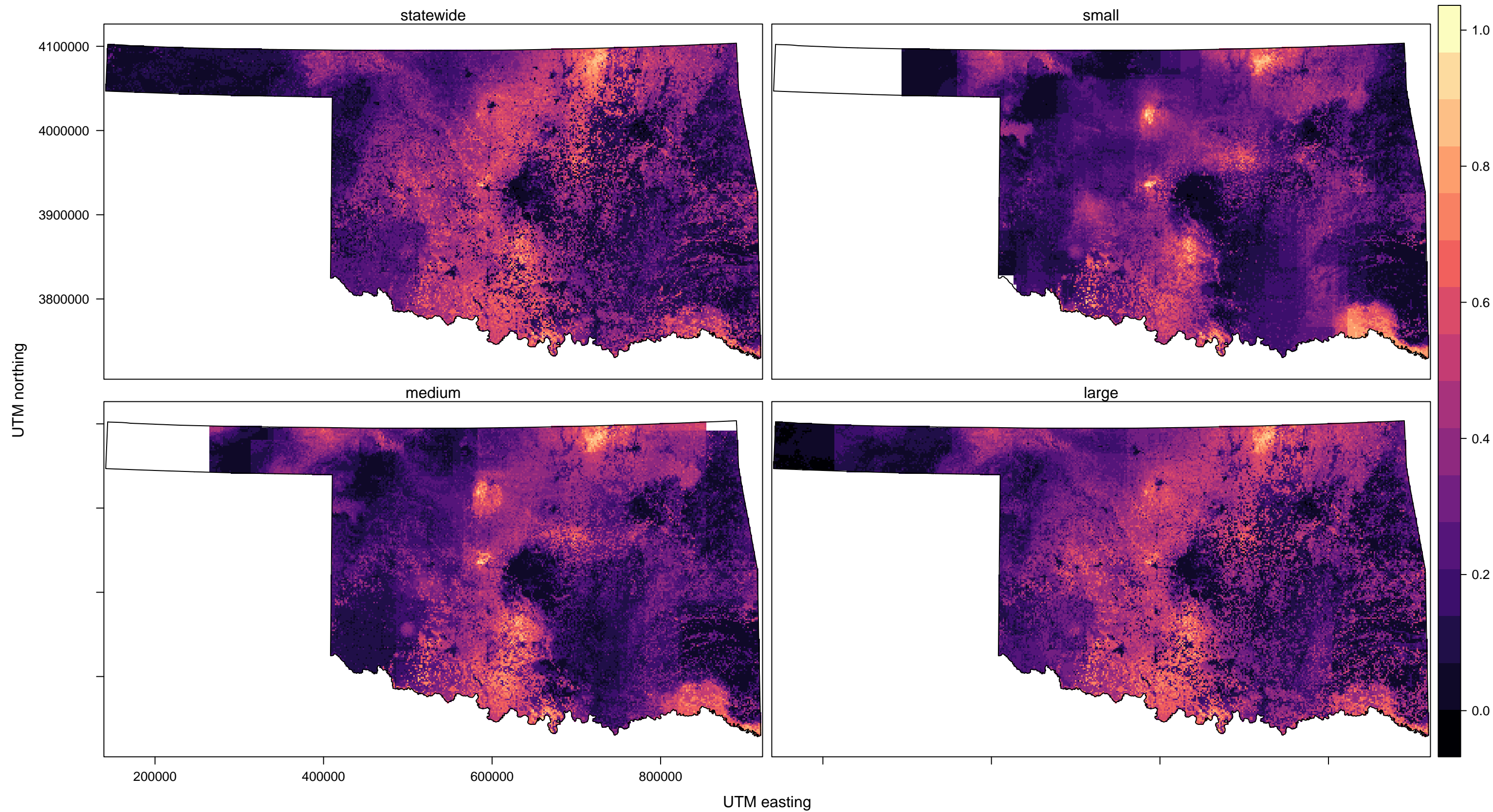

Supplement: Supplementary file 6 [file ECE3-8-12867-s006.pdf]

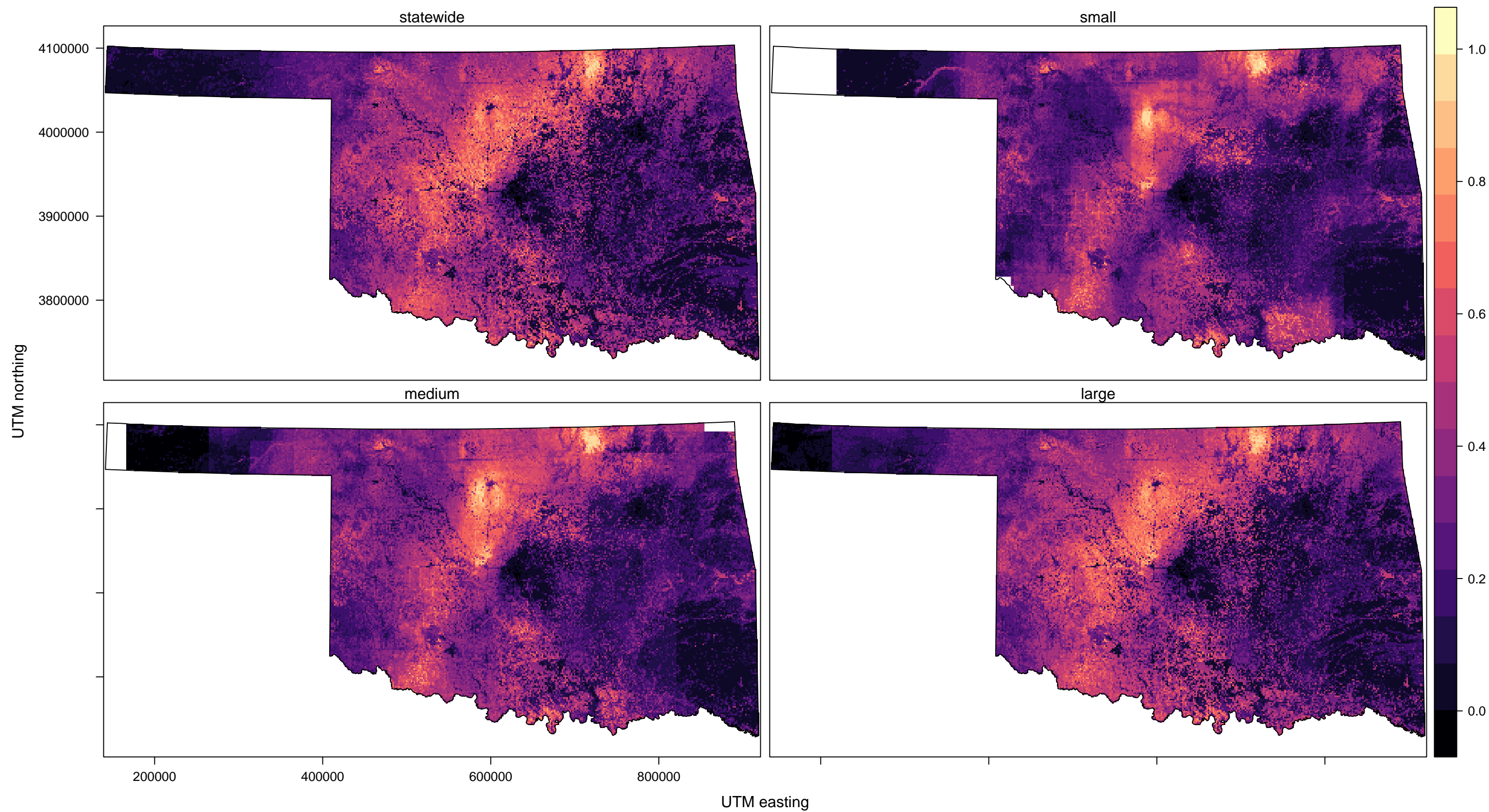

Supplement: Supplementary file 7 [file ECE3-8-12867-s007.pdf]
