# Supplementary material for: Varying dataset resolution alters predictive accuracy of spatially explicit ensemble models for avian species distribution
Source: Ecol Evol. 2018 Dec 6;8(24):12867–78. doi: 10.1002/ece3.4725 (PMC6308883; doi:10.1002/ece3.4725)
Supplement: Supplementary file 8 [file ECE3-8-12867-s008.docx]

# Supporting Information

Table S1. Predictors used in models.

| Predictor variable name | Definition | Source |
| --- | --- | --- |
| effort_length | Length of survey (km) | Survey data |
| effort_time | Duration of survey (hr) | Survey data |
| time_of_day | Time of survey | Survey data |
| conservation_easements_presenceabsence | Presence (1) or absence (0) of a conservation easement | (USDA/NRCS - National Geospatial Center of Excellence 2010) |
| conservation_easements_CalcArea | Area of the conservation easement in which a given pixel exists (acres) |  |
| nlcd_ok_utm14_okmask | NLCD2011 Landcover Classes | (USDA/NRCS - National Geospatial Center of Excellence 2011)  Neighborhoods modified from NLCD landcover classes. |
|  | NLCD 2.25 ha (5x5 cells) and 20.25 ha (15 x 15 cells) neighborhoods: proportion of neighborhood with the named land cover classes (values range from 0 to 1). Definitions described the land cover type and list the category numbers included in each neighborhood. |  |
| undevopenspace_5cell_okmask  undevopenspace_15cell_okmask | Undeveloped open space (11, 31, 71, 81, 82, 95) |  |
| openwater11_5cell_okmask  openwater11_15cell_okmask | Open water (11) |  |
| dev_openspace21_5cell_okmask  dev_openspace21_15cell_okmask | Developed open space (21) |  |
| dev_low22_5cell_okmask | Low intensity development (22) |  |
| dev_med23_5cell_okmask | Medium intensity development (23) |  |
| dev_high24_5cell_okmask | High intensity development (24) |  |
| barren31_5cell_okmask  barren31_15cell_okmask | Barren (31) |  |
| forest41to43_5cell_okmask | Forest (41, 42, 43) |  |
| scrub52_5cell_okmask | Scrub and shrubland (52) |  |
| grasslands71_5cell_okmask | Grasslands (71) |  |
| pasturehay81_5cell_okmask | Pasture and hay (81) |  |
| croplands82_5cell_okmask | Croplands (82) |  |
| woodywetlands90_5cell_okmask | Woody wetlands (90) |  |
| herbwetlands95_5cell_okmask | Herbaceous wetlands (95) |  |
| census_utm_30m | Human population density in number per km^2^ | (U.S. Department of Commerce/U.S. Census Bureau 2010) |
| bio1_12_OK | BIO1 = Annual Mean Temperature | Bioclim variables from Worldclim (Hijmans *et al.* 2005) |
| bio_12_OK | BIO2 = Mean Diurnal Range (Mean of monthly (max temp - min temp)) |  |
| bio3_12_OK | BIO3 = Isothermality (BIO2/BIO7) (* 100) |  |
| bio4_12_OK | BIO4 = Temperature Seasonality (standard deviation *100) |  |
| bio5_12_OK | BIO5 = Max Temperature of Warmest Month |  |
| bio6_12_OK | BIO6 = Min Temperature of Coldest Month |  |
| bio7_12_OK | BIO7 = Temperature Annual Range (BIO5-BIO6) |  |
| bio8_12_OK | BIO8 = Mean Temperature of Wettest Quarter |  |
| bio9_12_OK | BIO9 = Mean Temperature of Driest Quarter |  |
| bio10_12_OK | BIO10 = Mean Temperature of Warmest Quarter |  |
| bio11_12_OK | BIO11 = Mean Temperature of Coldest Quarter |  |
| bio12_12_OK | BIO12 = Annual Precipitation |  |
| bio13_12_OK | BIO13 = Precipitation of Wettest Month |  |
| bio14_12_OK | BIO14 = Precipitation of Driest Month |  |
| bio15_12_OK | BIO15 = Precipitation Seasonality (Coefficient of Variation) |  |
| bio16_12_OK | BIO16 = Precipitation of Wettest Quarter |  |
| bio17_12_OK | BIO17 = Precipitation of Driest Quarter |  |
| bio18_12_OK | BIO18 = Precipitation of Warmest Quarter |  |
| bio19_12_OK | BIO19 = Precipitation of Coldest Quarter |  |
